# High level expression of differentially localized BAG-1 isoforms in some oestrogen receptor-positive human breast cancers

**DOI:** 10.1038/sj.bjc.6690805

**Published:** 1999-11

**Authors:** M Brimmell, J S Burns, P Munson, L McDonald, M J O’Hare, S R Lakhani, G Packham

**Affiliations:** Ludwig Institute for Cancer Research, Imperial College of Medicine, St Mary’s Campus, Norfolk Place, London W2 1PG, UK; Department of Surgery, Ludwig Institute for Cancer Research/University College London, Breast Cancer Laboratory, London, UK; Department of Histopathology, University College London Medical School, London, UK; Virology and Cell Biology, Department of Medical Microbiology, Imperial College of Medicine, London, UK

**Keywords:** breast cancer, BAG-1, BCL-2, oestrogen receptor

## Abstract

Sensitivity to oestrogens and apoptosis are critical determinants of the development and progression of breast cancer and reflect closely linked pathways in breast epithelial cells. For example, induction of BCL-2 oncoprotein expression by oestrogen contributes to suppression of apoptosis and BCL-2 and oestrogen receptor (ER) are frequently co-expressed in tumours. BAG-1/HAP is a multifunctional protein which complexes with BCL-2 and steroid hormone receptors (including the ER), and can suppress apoptosis and influence steroid hormone-dependent transcription. Therefore, analysis of expression of BAG-1 in human breast cancer is of considerable interest. BAG-1 was readily detected by immunostaining in normal breast epithelial cells and most ER-positive tumours, but was undetectable or weakly expressed in ER-negative tumours. BAG-1 positive cells showed a predominantly cytoplasmic or cytoplasmic plus nuclear distribution of staining. A correlation between ER and BAG-1 was also evident in breast cancer derived cell lines, as all lines examined with functional ER expression also expressed high levels of BAG-1. In addition to the prototypical 36 kDa BAG-1 isoform, breast cancer cells expressed higher molecular weight isoforms and, in contrast to BCL-2, BAG-1 expression was independent of oestrogens. BAG-1 isoforms were differentially localized to the nucleus or cytoplasm and this was also independent of oestrogens. These results demonstrate a close association between BAG-1 and functional ER expression and suggest BAG-1 may be useful as a therapeutic target or prognostic marker in breast cancer. © 1999 Cancer Research Campaign


*British Journal of Cancer* (1999) **81**(5), 1042–1051
© 1999 Cancer Research Campaign
Article no. bjoc.1999.0805

### High level expression of differentially localized BAG-1 isoforms in some oestrogen receptor-positive human breast cancers

**M Brimmell[1], JS Burns[2,*], P Munson[3], L McDonald[1,†], MJ O'Hare[2], SR Lakhani[3] and G Packham[1,4]**

[1]Ludwig Institute for Cancer Research, Imperial College of Medicine, St Mary's Campus, Norfolk Place, London W2 1PG, UK; [2]Ludwig Institute for Cancer Research/University College London, Breast Cancer Laboratory, Department of Surgery, London, UK; [3]Department of Histopathology, University College London Medical School, London, UK; [4]Virology and Cell Biology, Department of Medical Microbiology, Imperial College of Medicine, London, UK

**Summary** Sensitivity to oestrogens and apoptosis are critical determinants of the development and progression of breast cancer and reflect closely linked pathways in breast epithelial cells. For example, induction of BCL-2 oncoprotein expression by oestrogen contributes to suppression of apoptosis and BCL-2 and oestrogen receptor (ER) are frequently co-expressed in tumours. BAG-1/HAP is a multifunctional protein which complexes with BCL-2 and steroid hormone receptors (including the ER), and can suppress apoptosis and influence steroid hormone-dependent transcription. Therefore, analysis of expression of BAG-1 in human breast cancer is of considerable interest. BAG-1 was readily detected by immunostaining in normal breast epithelial cells and most ER-positive tumours, but was undetectable or weakly expressed in ER-negative tumours. BAG-1 positive cells showed a predominantly cytoplasmic or cytoplasmic plus nuclear distribution of staining. A correlation between ER and BAG-1 was also evident in breast cancer derived cell lines, as all lines examined with functional ER expression also expressed high levels of BAG-1. In addition to the prototypical 36 kDa BAG-1 isoform, breast cancer cells expressed higher molecular weight isoforms and, in contrast to BCL-2, BAG-1 expression was independent of oestrogens. BAG-1 isoforms were differentially localized to the nucleus or cytoplasm and this was also independent of oestrogens. These results demonstrate a close association between BAG-1 and functional ER expression and suggest BAG-1 may be useful as a therapeutic target or prognostic marker in breast cancer. © 1999 Cancer Research Campaign

**Keywords**: breast cancer; BAG-1; BCL-2; oestrogen receptor

BAG-1 (also known as HAP; Zeiner et al, 1997) is a multifunctional protein which interacts with and modulates the activity of functionally diverse targets. BAG-1 was first identified as a BCL-2-binding protein that cooperated with BCL-2 to suppress apoptosis induced by staurosporine, Fas or cytotoxic T-lymphocytes in Jurkat cells (Takayama et al, 1995) and BAG-1 overexpression alone or in conjunction with BCL-2 has since been shown to contribute to suppression of apoptosis induced by diverse stimuli in a variety of cell types (Clevenger et al, 1997; Schulz et al, 1997; Takayama et al, 1997; Kullmann et al, 1998). BAG-1 also interacts with steroid hormone receptors, including the androgen, oestrogen and glucocorticoid receptors (Zeiner and Gehring, 1995; Froesch et al, 1998) and the retinoic acid receptor (Liu et al, 1998) and BAG-1 influences transcriptional activation and apoptosis induced by steroid hormones and retinoids (Froesch et al, 1998; Kullmann et al, 1998; Liu et al, 1998). BAG-1 binds some tyrosine kinase growth factor receptors (the hepatocyte growth factor and platelet derived growth factor receptors) and enhances the ability of these receptors to suppress apoptosis (Bardelli et al 1997), and can bind and activate the RAF-1 serine/threonine kinase (Wang et al, 1996). BAG-1 interacts with the human homologue of *Drosophila* seven in absentia (Siah) and inhibits p53-mediated growth arrest (Matsuzawa et al, 1998). Finally, BAG-1 binds directly and tightly to HSP/HSC70 heat shock proteins and modulates their chaperone activity (Hohfeld and Jentsch, 1997; Takayama et al, 1997; Zeiner et al, 1997). It is possible that binding to some BAG-1 partners, for example, the oestrogen receptor (ER), may be mediated by heat shock proteins, since HSP70 has been identified in ER complexes (Landel et al, 1994). Thus, BAG-1 potentially regulates several important cell growth control pathways.

The prototypical BAG-1 proteins in murine and human cells have molecular masses of approximately 32 kDa and 36 kDa respectively (Takayama et al, 1995, 1996; Packham et al, 1997). More recently, we and others identified novel higher molecular weight BAG-1 isoforms in human and mouse cells (Zeiner and Gehring, 1995; Packham et al, 1997; Takayama et al, 1998). Human and mouse cells express an approximately 50 kDa form, and an approximately 46 kDa form (also called RAP46; Zeiner and Gehring, 1995) has additionally been observed in human cells. These BAG-1/HAP isoforms have been referred to as p50, p46 and p36 BAG-1 (Packham et al, 1997) or as BAG-1L, BAG-1M and BAG-1 respectively (Takayama et al, 1998). The larger molecular

*Received 28 August 1998*
*Revised 10 November 1998*
*Accepted 25 November 1998*

*Correspondence to:* G Packham

*Present address: Centre for Proteome Analysis, Odense University, International Science Park Odense, Forskerparken 10B, DK-5230, Denmark
†Present address: Murdoch Institute, Royal Children's Hospital, Flemington Road, Parkville, Victoria 3052, Australia weight isoforms are generated by translation initiation at upstream codons and have unique N-terminal sequences relative to the major isoform (Packham et al, 1997; Takayama et al, 1998). The 50 kDa isoform is generated by initiation at a CUG codon whereas, like the prototypical BAG-1 isoform, the 46 kDa form initiates at a AUG codon. All BAG-1 isoforms contain a ubiquitin-like domain and varying numbers of a six amino-acid repeat (Takayama et al, 1995, 1998; Packham et al, 1997) although the functional significance of these motifs is not known. Two potential nuclear localization sequences (NLS) have been identified in BAG-1 isoforms. Mouse and human p50 isoforms uniquely contain a sequence which is very similar to the NLS of SV40 large T antigen and nucleoplasmin (Packham et al, 1997; Takayama et al, 1998). All human BAG-1 proteins also contain a potential bipartite NLS, but this sequence is relatively poorly conserved in mouse (Zeiner and Gehring, 1995; Packham et al, 1997). Although the major p36/p32 BAG-1 isoforms localize to the cytoplasm and p50 is predominantly present in the nucleus (Packham et al, 1997; Takayama et al, 1998), the roles of these potential NLS have not been addressed.

Regulation of cell proliferation and survival by oestrogens is a key component of growth control of normal and malignant breast epithelial cells, and ER status is an important prognostic and predictive marker of response to hormone therapy in human breast cancer. Oestrogens function primarily by binding to and activating the ER which is a ligand-dependent transcription factor. Following hormone depletion or treatment with anti-oestrogens, cells withdraw from the cell cycle and/or undergo cell death (Bardon et al 1987; Lippman and Dickson, 1989; Kyprianou et al, 1991; Wilson et al, 1995). Although there is some conflicting evidence, the cell death following hormone withdrawal appears to be due to activation of apoptosis (Kyprianou et al, 1991; Wilson et al, 1995). Thus, apoptosis and oestrogen-responsive are closely linked pathways in breast cancer. One pathway by which oestrogens may promote viability of breast cancer cells is via induction of BCL-2 since BCL-2 expression in MCF7 breast cancer cells is regulated by oestrogens and enforced BCL-2 expression suppresses MCF7 cell apoptosis (Texeira et al, 1995; Huang et al, 1997). This may explain the frequent co-expression of ER and BCL-2 in breast tumours (Bharagava et al, 1994; Leek et al, 1994; Silvestrini et al, 1994).

Since sensitivity of breast epithelial cells to apoptosis and oestrogens are closely linked and key determinants of breast cancer development and progression, BAG-1 may be important in breast cancer. For example, BAG-1 may cooperate with relatively low levels of BCL-2 to provide effective protection from apoptosis. BAG-1 may also influence the response of cells to oestrogens by direct effects on ER function. The aims of this study are to investigate the expression of BAG-1 isoforms in normal and malignant breast cells. We show that multiple BAG-1 isoforms are highly expressed in some breast cancers and, like BCL-2, their expression is often associated with positive ER status. However, expression and subcellular localization of BAG-1 isoforms are not regulated by oestrogens. BAG-1 may be an important prognostic marker or therapeutic target in breast cancer.

### MATERIALS AND METHODS

### Cell lines and cell culture

The breast cancer derived cell lines used in this study were obtained from ATCC, except SK-BR-3 and -7 cells which were from the New York (Sloane Kettering) branch of LICR, CAL-51 (Gionanni et al, 1990) which was obtained from the Dutrillaux Laboratory and GI-101 (Hurst et al, 1993) which was kindly forwarded by Prof CM Steel. The cell lines were maintained in Dulbecco's modified Eagle's medium (DMEM; Gibco/Life Technologies) supplemented with 10% fetal calf serum (FCS). For experiments requiring depletion of exogenous steroid hormones, cells were first washed thoroughly in phenol red-free DMEM and then cultured in phenol red-free DMEM supplemented with 10% charcoal stripped FCS for 4 days before stimulation with 10 nM 17β-estradiol (Sigma). Purified luminal or myoepithelial cells were obtained by immunomagnetic separation from clinical mammoplasty tissue (obtained from the Stephen Kirby Skin Bank) as previously described (Clarke et al, 1994). This procedure is based on the exclusive expression of epithelial membrane antigen (EMA) and CALLA (CD10) on luminal and myoepithelial cells respectively, and highly enriched cell preparations (> 98% purity) are isolated using superparamagnetic (MACS) beads. ICI 182 780 was obtained from Tocris (Bristol, UK) and was prepared as a 100 mM stock solution in dimethyl sulphoxide (DMSO).

### Tumour sections and immunostaining

All sections were dewaxed in xylene, taken to absolute alcohol (74OP) and endogenous peroxidase activity blocked with 3% hydrogen peroxide in methanol for 10 min. Sections were rinsed in tap water and transferred to a 15 lb per square inch pressure cooker containing 3 litres of boiling citrate buffer (pH 6.0). The slides were cooked at full pressure for 2 min and transferred to running tap water. The sections were blocked in normal goat serum (1/5 in Tris-buffered saline) for 10 min and incubated overnight at room temperature with the BAG-1 polyclonal at a final dilution of 1/1000 or for 1 h at room temperature with the ER antibody (clone 1DS) at a final dilution of 1/40. The primary antibodies were rinsed off in Tris-buffered saline and the slides transferred to a Dako TechMate instrument for detection. For BAG-1 staining, slides were incubated in ChemMate Link antibody (biotinylated goat anti-rabbit antibody) for 30 min and in ChemMate horse-radish peroxidase (HRP)-conjugated streptavidin for 30 min. ER staining was detected using the Dako strepAB complex/HRP Duet system. Slides were incubated in the link antibody and in the avidin–biotin complex for 35 min. Slides were visualized with diaminobenzidine, counterstained in Mayer's haematoxylin and mounted in synthetic mountant. Sections were processed in parallel but with the primary antibody omitted as a negative control in each experiment.

### Antibodies and immunoblotting

BAG-1 specific monoclonal and polyclonal antibodies were prepared using a fusion protein containing human p36 BAG-1 fused to the C-terminus of glutathione-S-transferase (GST) as an immunogen. Human BAG-1 sequences were amplified in a polymerase chain reaction (PCR) using primers V5827 (CGCGGATC-CGAGATGAATCGGAGCCAG) and T0459 (CCGGGATC-CTGCTACACCTCACTCGGCCAG) and plasmid p42 (Packham et al, 1997) as a template. The PCR product was digested using *Bam*HI and cloned into plasmid pGEX2TK (Pharmacia) which had been digested with the same restriction enzyme to give pGEX.BAG-1. BL21 *Escherichia coli* cells were transformed with pGEX.BAG-1, GST-BAG-1 expression induced using

**Table 1** Patient and tumour characteristics

| Tumour no. | Age | Histology | Grade[a] | ER status | Tumour size/mm | Nodal status |
|---|---|---|---|---|---|---|
| 96/8797 | 48 | MC | I | + | 32 | 0/13 |
| 96/9382 | 65 | IDC-NST | I | + | 11 | NR |
| 96/13497 | 50 | TUB | I | + | 15 | NR |
| 96/14922 | 58 | IDC-NST | I | + | 8 | NR |
| 96/9031 | 59 | IDC-NST | II | + | 21 | 3/15 |
| 97/1206 | 57 | MC | I | + | 15 | 0/18 |
| 96/10444 | 46 | IDC-NST | I | + | 30 | NR |
| 96/13909 | 57 | IDC-NST | I | + | 12 | NR |
| 96/7332 | 57 | IDC-NST | I | + | 12 | NR |
| 96/11196 | 51 | IDC-NST | I | + | 21 | 0/12 |
| 96/13658 | 45 | IDC-NST | II | – | 22 | 4/8 |
| 96/15630 | 41 | IDC-NST | III | – | 4 | 4/17 |
| 97/2380 | 49 | IDC-NST | III | – | 14 | 0/16 |
| 96/9041 | 47 | IDC-NST | III | – | 29 | 0/23 |
| 96/5863 | 62 | IDC-NST | III | – | 24 | 0/24 |
| 96/9953 | 26 | AM | III | – | 30 | 0/7 |
| 96/9827 | 84 | IDC-NST | III | – | 40 | NR |
| 97/12680 | 60 | IDC-NST | III | – | 23 | 4/10 |
| 97/3377 | 31 | IDC-NST | II | – | 11 | 0/10 |
| 97/4880 | 37 | IDC-NST | II | –[b] | 3 | NR |

MC, mucinous carcinoma; IDC-NST, infiltrating ductal carcinoma, no special type; TUB, tubular carcinoma; AM, atypical medullary carcinoma; NR, no axillary resection performed; +, ER expressed in >10% of tumour cells; –, ER expressed in <10% of tumour cells. [a]Modified Bloom and Richardson Grading System. [b]Tumour 97/4880 comprised ER-negative IDC and largely ER-negative carcinoma in situ with focal ER positivity.

isopropyl-β-thiogalactopyranoside and purified using glutathione sepharose 4B beads (Pharmacia).

The GST-BAG-1 fusion protein was used to repeatedly immunize female New Zealand White rabbits and BALB/c mice for production of polyclonal and monoclonal antibodies respectively. Splenocyte fusions were made as previously described using SP2 cells (Fredersdorf et al, 1996) and hybridomas single-cell cloned by limiting dilution. Clone 3.10 G3 E2 was selected for immunoblotting analysis of BAG-1 expression.

Western blotting was performed as previously described (Packham et al, 1997). The BAG-1 monoclonal antibody (clone 3.10 G3 E2) was used as undiluted hybridoma supernatant and the BAG-1 polyclonal antibody was used at a final dilution of 1/200. The mouse anti-BCL-2 antibody (C124, Dako) and rabbit anti-ER antibody (HC-20, Santa Cruz Biotechnology) were used at a final concentrations of 1 μg $ml^{-1}$. The green fluorescent protein specific rabbit antibody (Clontech) was used at a final dilution of 1/2000. The F1βATPase specific rabbit antisera was a kind gift of Dr W Neupert (Ludwig-Maximilians Universitat, Munich, Germany) and was used at a dilution of 1/2000. The histone specific mouse monoclonal antibody (Chemicon) was used at a final dilution of 1/2000. The HSC70 specific goat antibody (K-19, Santa Cruz Biotechnology) was used at a final concentration of 0.6 μg $ml^{-1}$. The second layer antibodies were donkey HRP-conjugated anti-rabbit Ig and sheep HRP-conjugated anti-mouse Ig (both Amersham) at final dilutions of 1/5000, or rabbit HRP-conjugated anti-goat Ig (Santa Cruz Biotechnology) at a final dilution of 1/2000. BAG-1 isoforms were generated by in vitro translation using plasmids p42, p276 and p289 as previously described (Packham et al, 1997).

### Cell fractionation

Nuclear, membrane and cytosolic fractions were obtained by hypotonic lysis and differential centrifugation. Cells were washed twice in phosphate-buffered saline and resuspended in 1 ml of hypotonic lysis buffer (10 mM Tris–HCl, pH 7.4; 3 mM calcium chloride; 2 mM magnesium chloride; 100 mM phenylmethyl-sulphonyl fluoride; 1 mM aprotinin; 1 mM leupeptin; 1 mM $Na_2VO_3$; 1 mM NaF). After incubation on ice for 10 min, cells were lysed using a dounce homogenizer. The homogenate was centrifuged at 500 ***g*** for 5 min at 4°C and supernatant removed. The pellet was resuspended in 0.5 ml of lysis buffer and centrifuged as before. The two supernatants were combined and the pellet retained as the nuclear pellet. The supernatant was centrifuged at 100 000 ***g*** for 1 h at 4°C using a TLA45 rotor (Beckman). The resultant pellet was retained as the membrane fraction and the supernatant as the cytosolic fraction. Pellets were resuspended in a volume of lysis buffer equal to the final volume of cytosol (1.5 ml) and disrupted by brief sonication. A lactate dehydrogenase (LDH) assay (Sigma Diagnostics) was used to monitor cytosolic contamination of nuclear and membrane fractions.

### Green fluorescent protein fusion constructs

The fluorescent fusion proteins were based upon the EGFP-N1 plasmid (Clontech) which expresses a modified green fluorescent protein (EGFP) under transcriptional control of cytomegalovirus immediate early enhancer regulatory sequences. To generate p36EGFP (which contains the complete p36BAG-1 coding sequence fused in frame to the 5′ of EFGP sequences), plasmid p42 (Packham et al, 1997) was amplified by PCR using primers Y0075 (CCGGAATTCACCATGAATCGGAGCCAGGAGGTG) and Y0076 (CGCGGATCCTCGGCCAGGGCAAAGTTTG). To generate p50NtEGFP (which contains the unique p50BAG-1 N-terminal sequences which are absent from p36BAG-1 fused in frame to the 5′ end of EFGP sequences), plasmid p42 (Packham et al, 1997) was amplified using primers W8444 (CCGGAATTCACCATGGCTCAGCGCGGGGGG) and W7882

 *© 1999 Cancer Research Campaign*

Table 2 BAG-1 immunostaining in ER-positive and ER-negative breast tumours

| Tumour no. | Associated normal tissues | Associated benign lesions | DCIS | IDC |
|---|---|---|---|---|
| ER-positive tumours | | | | |
| 96/8797 | + | | + | + |
| 96/9382 | + | | ± | ± |
| 96/13497 | + | ± (AC) | + | + |
| 96/14922 | + | + (H+SA) | + | + |
| 96/9031 | + | | ± | ± |
| 97/1206 | + | | | + |
| 96/10444 | + | | + | + |
| 96/13909 | + | | ± | ± |
| 96/7332 | | | | + |
| 96/11196 | + | | ± | – |
| ER-negative tumours | | | | |
| 96/13658 | + | | | ± |
| 96/15630 | + | | ± | ± |
| 97/2380 | + | | | ± |
| 96/9041 | + | + (H) | | – |
| 96/5863 | + | | | ± |
| 96/9953 | + | | | – |
| 96/9827 | | | | ± |
| 97/12680 | ± | | | ± |
| 97/3377 | + | | – | – |
| 97/4880 | + | | –/+[a] | ± |

DCIS, ductal carcinoma in situ: IDC, inflitrating ductal carcinoma; AC, apocrine cyst; H, hyperplasia; SA, sclerosing adenosis; –, no detectable expression; +, positive (>90% cells intense staining); ±, most cells negative (focal staining [<10% positive cells] or weakly positive at tumour edge). Blanks indicate material not present in section. [a]Tumour 97/4880 comprised heterogenous DCIS largely comprised of ER/BAG-1 negative cells with foci of ER/BAG-1 positivity.

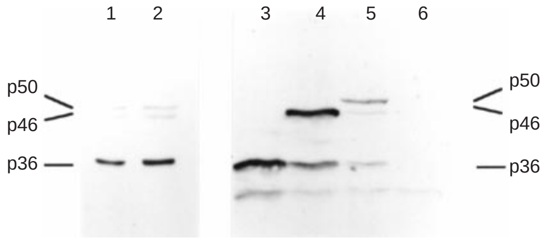

**Figure 1** Characterization of BAG-1-specific polyclonal antibody. Immunoblot analysis of BAG-1 isoforms in human breast cancer cell lines and generated in vitro using rabbit reticulocyte lysates. Lanes 1 and 2, RIPA lysates (20 μg) prepared from T47D and ZR-75-1 human breast cancer cell lines respectively. Lanes 3–6 are BAG-1 proteins made in reticulocyte lysates programmed with plasmids p289 (lane 3), p276 (lane 4) and p42 (lane 5), or unprogrammed lysate as a negative control (lane 6). The p36, p46 and p50 BAG-1 isoforms are indicated. The fastest migrating band in lanes 3–6 corresponds to endogenous rabbit BAG-1

(CGCGGATCCTCTTCGCCCTGGGTCGCC). The amplification products were digested with *Eco*RI and *Bam*HI and cloned into plasmid EGFP-N1 which had been digested with the same enzymes. MCF7 and SAOS2 osteosarcoma cells were transfected using the SuperFect reagent (Qiagen) according to the manufacturers' instructions and analysed for EGFP expression by fluorescence microscopy and immunoblotting after 24 h.

### RESULTS

### BAG-1 immunostaining in normal breast epithelia and breast tumours

We prepared a BAG-1-specific polyclonal antibody which was suitable for staining of archival paraffin-embedded material. The specificity of the antibody for BAG-1 isoforms was confirmed by immunoblot analysis (Figure 1). When tested against lysates prepared from human breast cancer cell lines (lanes 1 and 2), the antisera recognized the three previously described human BAG-1 isoforms (p36, p46 and p50 BAG-1) and its reactivity was identical to other BAG-1 antibodies (c.f. Figure 4). Moreover, the antibody specifically recognised all three BAG-1 isoforms generated by in vitro translation of the human BAG-1cDNA (Packham et al, 1997) (lanes 3–6).

Twenty cases of breast carcinomas were analysed for BAG-1 expression using the BAG-1 specific polyclonal antibody (Table 1). We randomly selected ten ER-positive cases and ten ER-negative cases from the archives in histopathology at University

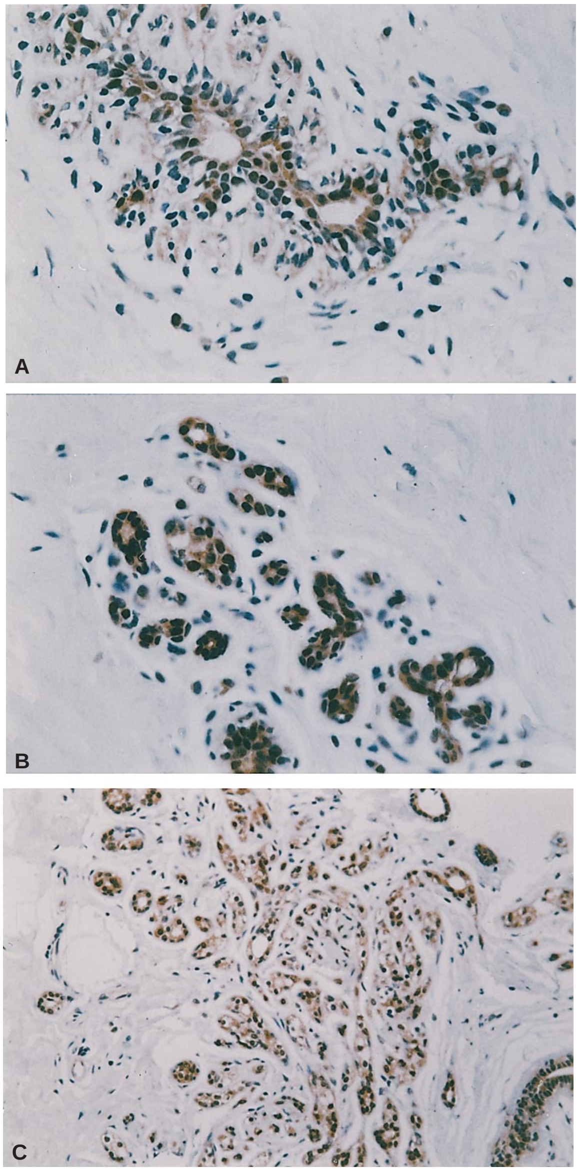

**Figure 2** BAG-1 immunostaining in normal and benign hyperproliferative breast epithelia. Representative examples of BAG-1 immunostaining in uninvolved epithelia associated with tumours 97/12680 **(A)** and 96/14922 **(B)** and sclerosing adenosis associated with tumour 96/14922 **(C)**

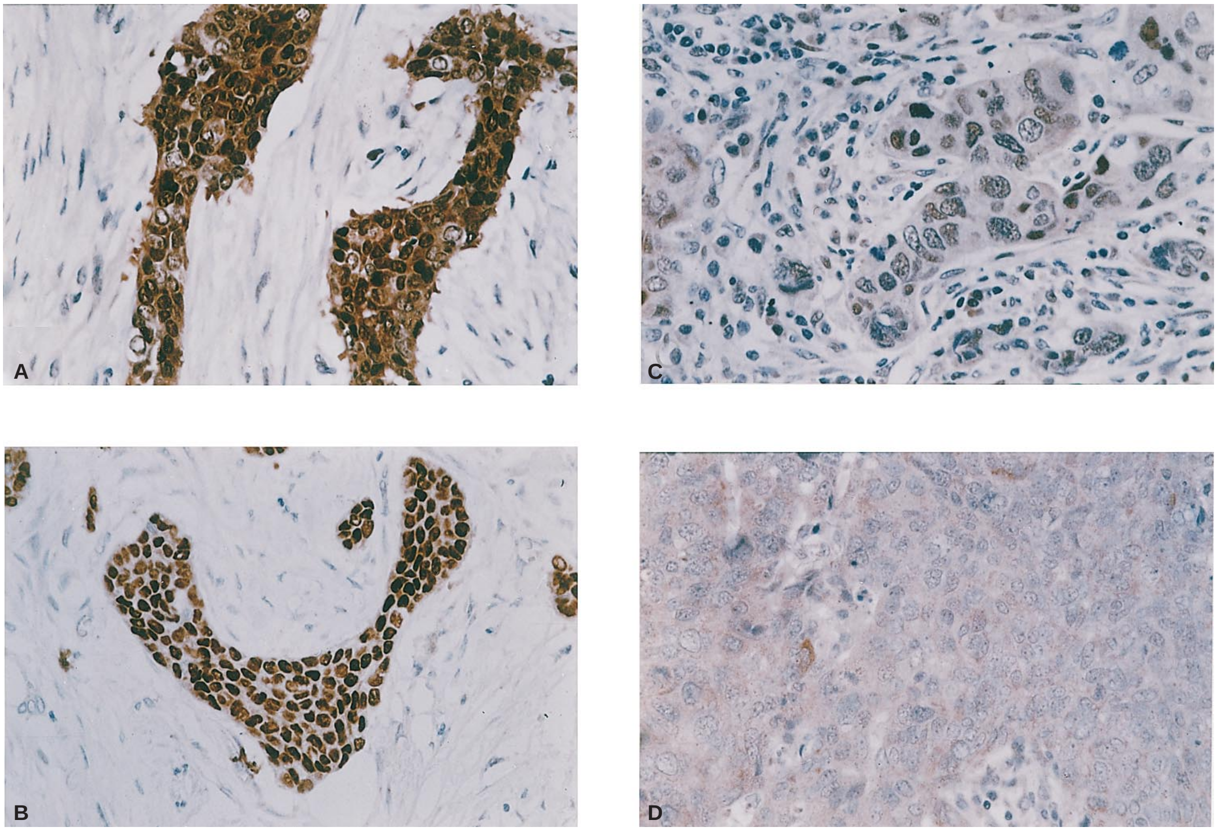

**Figure 3** BAG-1 is readily detectable in ER-positive, but not ER-negative, breast carcinomas. Representative example of **(A)** BAG-1 and **(B)** ER expression in an ER-positive tumour (96/14922) and **(C)** region of weak/focal BAG-1 expression and **(D)** lack of ER expression in an ER-negative tumour (97/12680)

College London. In addition to the infiltrating ductal carcinoma (IDC) component of these tumours, BAG-1 immunostaining was determined in ductal carcinoma in situ (DCIS), associated benign lesions and adjacent normal breast epithelia. Similar immunostaining results were obtained using BAG-1-specific monoclonal antibodies and immunostaining of the polyclonal antibody was specifically ablated by incubation with GST-BAG-1, but not GST (data not shown).

BAG-1 was detected in 18/18 (100%) of normal breast tissues examined. BAG-1 was readily detected in luminal cells, whereas staining was less intense or absent in myoepithelial cells, and BAG-1 was undetectable in surrounding lymphocytes and stromal cells (Figure 2 A,B). A recent survey of BAG-1 expression in normal human tissues showed a similar pattern of BAG-1 expression predominantly in luminal epithelial cells of the breast (Takayama et al, 1998). Cells showed a predominantly cytoplasmic or cytoplasmic plus nuclear distribution of BAG-1 staining. BAG-1 expression was also detected in associated benign lesions, including sclerosing adenosis (Figure 2C). A summary of BAG-1 expression is presented in Table 2.

BAG-1 was detected in 9/10 (90%) of ER positive tumours (Figure 3 A,B). 6/10 of cases showed very intense staining in essentially all tumour cells, whereas in the remaining cases, BAG-1 was detectable but staining was weaker and/or focal (3/10 cases) or undetectable (1/10 cases). In most cases, both DCIS and IDC components were positive; one case was positive for BAG-1 expression in DCIS only. Again tumour cells showed a predominantly cytoplasmic or cytoplasmic plus nuclear distribution and in some cases, BAG-1 expression appeared to be somewhat stronger in tumour cells relative to uninvolved adjacent epithelia.

In contrast to the ER-positive tumours, BAG-1 was less readily detectable in the ten ER-negative cases (Figure 3 C, D). BAG-1 was completely undetectable in 3/10 cases (but was detected in surrounding normal tissue) and in the other seven cases, BAG-1 expression was either weakly positive only at the tumour margin (two cases) or weakly expressed only at focal points (< 10% positive cells; five cases). No ER-negative tumour stained as strongly as the intense staining observed in most ER-positive tumours. Interestingly, one tumour (97/4880) contained DCIS which was largely comprised of ER-negative cells but with focal ER positivity. BAG-1 expression was restricted to ER-positive cells (Table 2). Therefore, BAG-1 is expressed at high levels in normal breast epithelia and in some breast tumours, and its expression is often correlated with positive ER status.

### BAG-1 isoform expression in breast cancer cell lines

To further explore the pattern of BAG-1 isoform expression in breast cancer cells, we used a BAG-1-specific monoclonal antibody to analyse a panel of cell lines derived from breast cancers and preparations of normal breast epithelial cells. BAG-1 isoforms were detected in all cell lines and preparations at varying levels (Figure 4 A, B). BAG-1 was expressed at very low levels in CAL51, CAMA1, SK-BR-7 and BT20 cells and at the highest level in ZR-75-1, MCF7, MDA-MB-453 and GI-101 cells. Consistent with results of immunostaining, BAG-1 expression was

 *© 1999 Cancer Research Campaign*

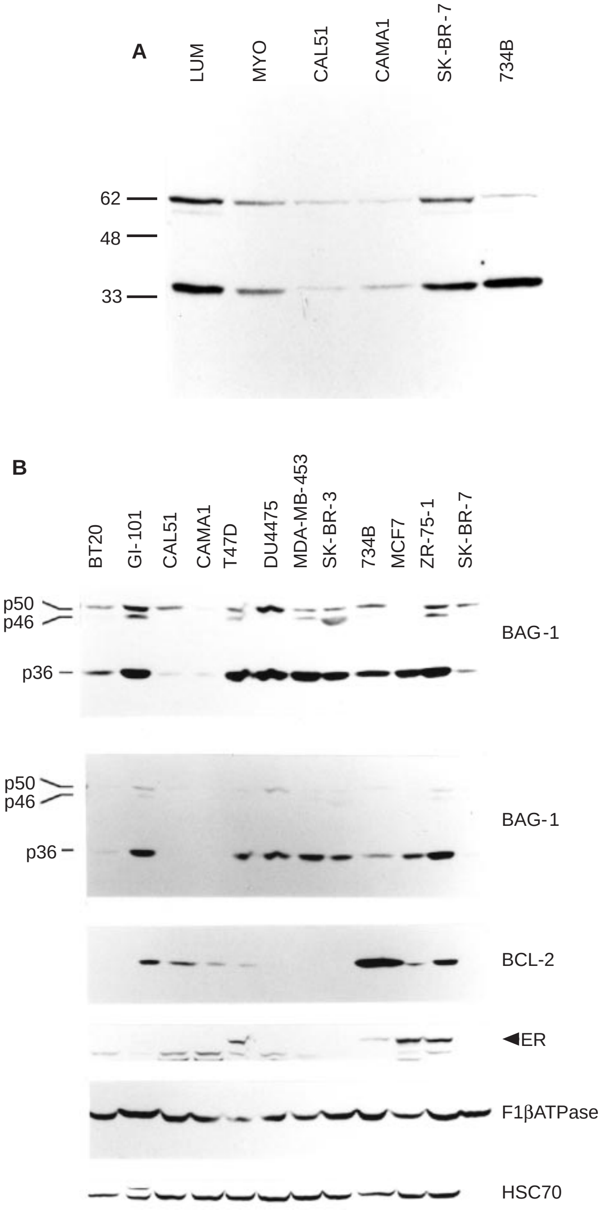

**Figure 4** **(A)** Expression of BAG-1 isoforms in preparations of primary luminal (LUM) and myoepithelial (MYO) cells derived from normal breast. **(B)** Expression of BAG-1 isoforms, BCL-2, ER, HSC70 and F1βATPase (as a loading control) in a panel of breast cancer-derived cell lines. SK-BR-7 cells were not tested for HSC70 expression. The lower molecular weight bands detected by the ER antibody in this experiment are due to cross-reactions and were not detected in other experiments. Two exposures of the BAG-1 blot are shown to more clearly demonstrate expression of p46 and p50 isoforms and low level expression of p36 BAG-1 in CAL51 and CAMA1 cells lower in normal myoepithelial cells than in luminal cells and BAG-1 expression in normal cells was low relative to many cell lines.

Multiple BAG-1 isoforms were detected in each sample (Figure 4). p36 BAG-1 was generally the most abundant isoform and its levels differed significantly between cell lines. Expression of the larger isoforms was generally lower and more consistent between cell lines relative to variations in p36 BAG-1. However, in cell lines such as BT20, CAL51 and CAMA1 with low levels of p36, the larger isoforms were at least as abundant as p36 BAG-1. Of the larger isoforms, some cell lines expressed only p50 with only very low level of p46, whereas others expressed approximately equal amounts of p46 and p50.

We also examined expression of the BAG-1 binding proteins, BCL-2, ER and the constitutive 70 kDa heat shock protein, HSC70, in the panel of cell lines (Figure 4B). BCL-2 expression was also variable in the cell lines and 734B, ZR-75-1, GI-101 and CAL51 had the highest levels of expression. ER was readily detected in four cell lines, T47D, 734B, MCF7 and ZR-75-1. Consistent with previous reports (Lippman et al, 1976; Engel et al, 1978; Chalbos et al, 1982; Coradini et al, 1991), the ER was functional in each of the lines with high level ER expression since oestrogen induced S phase entry and/or activated ER-dependent transcription in these cells (data not shown). Therefore, similar to the tumour sections, there was a correlation between ER status and BAG-1 expression; all cell lines with high level functional ER expression also expressed high levels of BAG-1 isoforms. HSC70 was readily detectable in all lines and expression levels did not vary significantly.

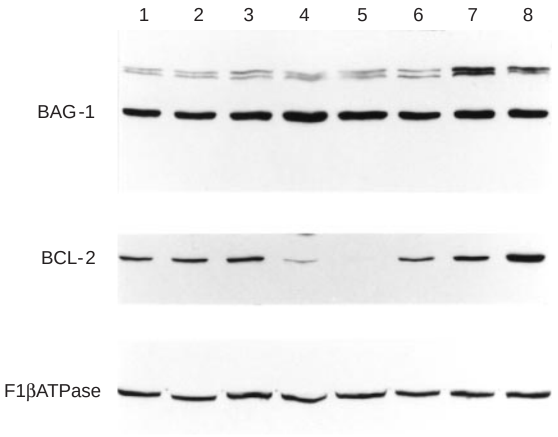

**Figure 5** BCL-2, but not BAG-1 expression is regulated by oestrogens in MCF7 cells. MCF7 cells were cultured in standard growth conditions (DMEM with phenol red and complete FCS) (lane 1), standard growth medium plus carrier (DMSO) for 2 or 4 days (lanes 2 and 3), standard growth medium plus ICI 182 780 (50 nM) for 2 or 4 days (lanes 4 and 5) or were cultured in hormone depleted conditions (phenol red-free DMEM plus charcoal-stripped FCS) for 5 days (lane 6) and then treated with 10 nM 17β-oestradiol for 2 or 5 days (lanes 7 and 8). Cells were analysed for expression of BAG-1, BCL-2 and F1βATPase (as a control) by immunoblotting

### Effects of oestrogen on BAG-1 and BCL-2 expression in MCF7 cells

One explanation for the correlation between BAG-1 and ER expression in tumours and cell lines was that BAG-1 expression is oestrogen-responsive. Indeed, BCL-2 is regulated by oestrogens in breast cancer cells (Texeira et al, 1995; Huang et al, 1997) and BAG-1 and BCL-2 are co-regulated in some systems (Adachi et al, 1996). We therefore examined effects of modulating oestrogen signalling on BAG-1 and BCL-2 expression in oestrogen-responsive MCF7 cells. We first analysed cells grown in normal conditions (phenol red containing medium and complete FCS) and cells additionally exposed for 2 or 4 days to the pure anti-oestrogen ICI 182 780 (Wakeling et al, 1991). Consistent with previous reports (Texeira et al, 1995; Huang et al, 1997), exposure of MCF7 cells to ICI 182 780 significantly down-regulated BCL-2 expression

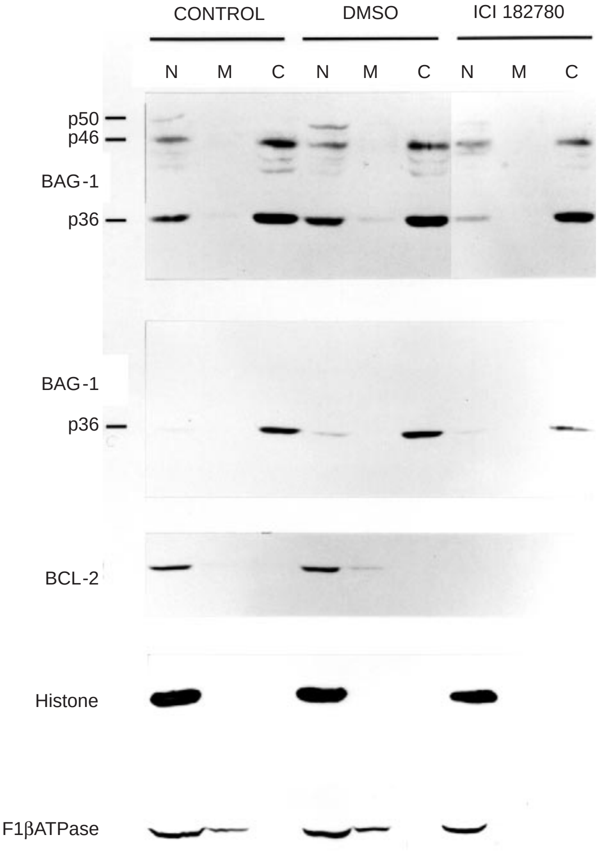

**Figure 6** Subcellular localization of BAG-1 isoforms in breast cancer cells. MCF7 cells were cultured in normal conditions (DMEM containing phenol red and complete FCS; control), normal conditions plus 0.05% DMSO or normal conditions plus ICI 182 780 (50 nM). Cells were collected after 2 days and fractionated into (N) nuclear, (M) heavy membrane and (C) cytosolic fractions by differential centrifugation. Equal volumes of each fraction were analysed by immunoblotting using BAG-1, BCL-2, histone (marker for nuclei) and F1βATPase (marker for mitochondria) specific antibodies. Assays for the cytosolic marker LDH were performed on each fraction and revealed that less than 20% of total LDH activity was contaminating the nuclear or membrane fractions. Two exposures of the BAG-1 immunoblot are shown to more clearly illustrate localization of p46 and p50 BAG-1 isoforms

(Figure 5). By contrast, BAG-1 expression was not altered. We also analysed cells which had been deprived of exogenous steroid hormones (cultured in medium lacking phenol red and supplemented with charcoal stripped FCS for 5 days) and then restimulated for 2 or 5 days with 17β-oestradiol. Under these conditions, BCL-2 expression was significantly elevated after 5 days, whereas BAG-1 expression was not altered. Therefore, the steady-state expression levels of BAG-1 isoforms are not coordinately regulated with BCL-2, and their expression is independent of oestrogen. Similar results were obtained using oestrogen-responsive T47D cells (data not shown).

### Analysis of the subcellular localization of BAG-1 isoforms

We analysed the localization of BAG-1 isoforms using differential centrifugation and green fluorescent protein (GFP) fusion proteins to determine whether they were differently localized in human breast cancer cells and whether this was regulated by oestrogens.

MCF7 cells were cultured in normal growth conditions and subcellular fractions prepared by differential centrifugation. Lysis was performed in the absence of detergents which have previously been shown to remove BCL-2 family proteins from membranes and might alter apparent localization of BAG-1 proteins. The 36 kDa isoform was predominantly present in the cytosol (Figure 6, second panel). The relatively small amount of p36 BAG-1 detected in the nuclear fraction (Figure 6) was consistent with the degree of cytosolic contamination as measured using LDH assays suggesting that p36 BAG-1 is exclusively present in the cytosol in MCF-7 cells. The 50 kDa isoform localized to the cell nucleus, whereas the 46 kDa isoform was present in both cytosolic and nuclear fractions. Although the nuclear fraction was heavily contaminated with mitochondria (presumably a result of the necessity to avoid detergents in the fractionation procedure), p50 and p46 were not detected in mitochondrial fractions (despite presence of F1βATPase reactivity) and their apparent localization to nuclei is likely to be real and not a result of mitochondrial contamination of these fractions. As previously described, BCL-2 localized to nuclear/membrane fractions (Reed, 1997).

We transfected MCF7 cells with expression plasmids encoding BAG-1/GFP fusion proteins to study localization in intact cells. The p50 BAG-1 isoform is unique in that its N-terminus contains a nuclear localization motif absent in other isoforms (Packham et al, 1997). To determine whether the unique N-terminus of p50 BAG-1 was sufficient to direct nuclear localization, we overexpressed a GFP fusion protein containing just these sequences. Whereas, the parental GFP localized in both the cell nucleus and cytosol (Figure 7), the p50 Nt-GFP protein was almost entirely localized to the cell nucleus and was focused at subdomains consistent with nucleoli. Therefore, the unique N-terminal sequences present in p50 BAG-1 are sufficient to confer nuclear localization. Localization of a fusion protein containing the p36 BAG-1 portion of BAG-1 was essentially identical to GFP. Western analysis demonstrated expression of GFP fusion proteins of the expected molecular masses in transfected cells (Figure 7D).

To test whether localization of BAG-1 isoforms was regulated by oestrogens, we fractionated MCF7 cells maintained in the presence or absence of ICI 182 780. The localization of BAG-1 isoforms was not altered by these conditions (Figure 6). Similarly, the distribution of endogenous BAG-1 isoforms was not altered by culture in hormone depleted conditions or hormone depleted condition plus 17β-oestradiol and the distribution of GFP/BAG-1 fusion proteins in transfected cells was also independent of culture conditions (data not shown). Therefore, although BAG-1 isoforms are differentially localized in breast cancer cells, this is independent of oestrogens.

### DISCUSSION

Here we have examined expression and regulation of BAG-1 isoforms in primary breast tumours and cell lines. Since BAG-1 interacts with BCL-2 and ER, and may influence sensitivity of breast cancer cells to apoptosis and steroid hormones, analysis of BAG-1 in breast cancer is of significant interest. BAG-1 was readily detected in normal breast epithelia and most ER-positive tumours. By contrast, BAG-1 expression was either undetectable or weak and/or focal in ER-negative tumours. Thus, high levels of BAG-1 expression are often associated with ER positivity in breast tumours. It is not clear whether tumours with no BAG-1 detectable by immunostaining are truly negative for BAG-1 expression. The expression of BAG-1 in all breast cancer cell lines (albeit at

 *© 1999 Cancer Research Campaign*

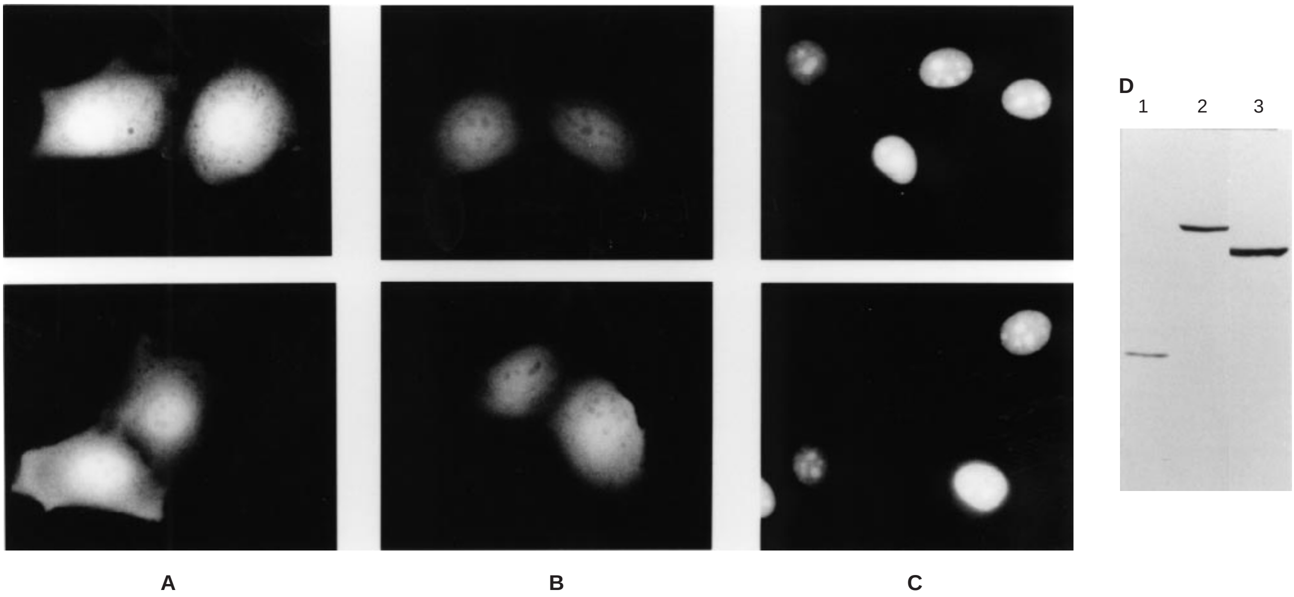

**Figure 7** Analysis of BAG-1 localization using green fluorescent fusion proteins. MCF7 cells maintained in DMEM (with phenol red and complete FCS) were transfected with plasmids expressing **(A)** EGFP, **(B)** p36BAG-1 fused to EGFP or **(C)** p50 unique N-terminal sequences fused to EGFP. After 24 h, cells were examined directly by fluorescence microscopy. **(D)** Immunoblot analysis using anti-GFP antibody of EGFP proteins in transiently transfected SAOS2 cells. Lane 1, EGFP; lane 2, p36BAG-1/EGFP; lane 3, p50Nt/EGFP

relatively low levels in some cases) suggests that BAG-1 is expressed in apparently BAG-1-negative tumours but below the level of detection of immunostaining. Regardless, there are clearly significant differences in expression of BAG-1 between individual breast tumours and this may be important. It is also possible that differences in subcellular distribution of BAG-1 immunostaining may be significant.

All normal and breast cancer cells expressed multiple BAG-1 isoforms and, similar to other cell types (Packham et al, 1997; Takayama et al, 1998) these are differently localized in MCF7 cells. p36 BAG-1 was the predominant isoform in most cells, although in some cell lines with low levels of p36, the higher molecular weight isoforms were also relatively abundant. Consistent with the presence of a sequence very similar to the nuclear localization sequence of SV40 large T antigen (Packham et al, 1997), the p50 isoform localized to the nucleus and analysis of GFPs suggested it was concentrated in subdomains consistent with nucleoli. The p36 isoform localized to the cytosol, whereas the p46 isoforms appeared to be distributed between cytoplasmic and nuclear fractions. A GFP fusion protein containing the p36 BAG-1 isoform was not distributed differently to GFP. Therefore, whereas the N-terminal NLS present only in p50 BAG-1 is sufficient to direct nuclear localization, the potential bipartite NLS present in all human BAG-1 isoforms (Zeiner and Gehring, 1995) is not, at least in these settings.

It is well established that BCL-2 expression strongly correlates with ER status (Bharagava et al, 1994; Leek et al, 1994; Silvestrini et al, 1994) and one would therefore predict a similar correlation between expression of BCL-2 and BAG-1. Consistent with this, all cell lines with functional ER co-expressed high levels of BAG-1 and BCL-2. BAG-1 and BCL-2 may therefore act as a couple in ER-positive breast cancer cells and cooperate in regulation of apoptosis. There is, however, a significant difference between expression of BCL-2 and BAG-1 in breast epithelial cells, since whereas the association between ER and BCL-2 may be explained by positive effects of oestrogens on BCL-2 expression (Texeira et al, 1995; Huang et al, 1997), BAG-1 is not coordinately regulated with BCL-2 by oestrogens and both the steady-state expression and subcellular localization of BAG-1 appears to be independent of oestrogens. Some ER-negative cell lines also had high expression of BCL-2 and/or BAG-1 suggesting a complex mode of regulation.

A key question that emerges from these studies is what is the functional significance of BAG-1 in normal and malignant breast cells? BAG-1 is multifunctional and the function of the individual isoforms may depend on both cell context and expression level. Positive effects of p50 BAG-1 isoforms on androgen receptor-dependent transcription and negative effects of p46 BAG-1 on glucocorticoid receptor-dependent transcription have been described (Froesch et al, 1998; Kullmann et al, 1998). Therefore, BAG-1 isoforms may also contribute to or modulate ER responsiveness of breast epithelial cells, possibly via effects on 70-kDa heat shock proteins which are detected in ER complexes (Landel et al, 1994). High level BAG-1 expression in ER-positive tumours may potentiate or limit activation of ER by hormone and therefore be important in determining the level of ER functionality. BAG-1 may also modulate the response of ER complexes to anti-oestrogens. To data, effects of BAG-1 on steroid hormone receptors have been limited to the higher molecular weight isoforms (Froesch et al, 1998; Kullmann et al, 1998), and the major BAG-1 isoform may play a distinct function. It is also possible that BAG-1 influences other steroid hormone receptors in breast cancers. For example, the glucocorticoid receptor was expressed in all cells examined (data not shown). However, the close correlation between BAG-1 and ER expression perhaps suggests that the ER is the most likely receptor target of BAG-1 function in this setting. HSC70 is a direct binding partner of BAG-1 and may mediate BAG-1 activities (Hohfeld and Jentsch, 1997; Takayama et al, 1997; Zeiner et al, 1997). However, HSC70 expression was invariant in the panel of breast cancer cell lines.

© 1999 Cancer Research Campaign

As mentioned above, BAG-1 may also function by directly influencing apoptosis of breast cancer cells in a BCL-2-dependent manner. BCL-2 proteins are thought to function, at least in part, by inserting into membranes and contributing to the formation or regulation of channels. It has been hypothesized that BAG-1 may alter BCL-2 function by acting on heat shock proteins which may facilitate conformational changes of BCL-2 itself or proteins which might pass through the BCL-2-dependent channels (Reed, 1997). Thus, breast cancer cells may be effectively protected from apoptosis by a relatively low level of BCL-2 in conjunction with significant BAG-1 expression. In addition, BAG-1 binds and modulates function of hepatocyte growth factor receptor (Bardelli et al, 1997) which may also be important in breast carcinogenesis (Tuck et al, 1996; Beviglia et al, 1997) and represent another potential target of BAG-1 in breast cancer.

The expression of BAG-1 was higher in some cell lines than in preparations of normal breast epithelial cells. In some ER-positive cases, BAG-1 staining in tumour cells was more intense than in surrounding normal cells. Overexpression of BAG-1 may therefore contribute to development of some ER-positive breast cancers. Conversely, BAG-1 expression was often reduced in ER-negative tumour cells relative to normal cells. A recent report also noted overexpression of BAG-1 relative to normal cells in some tumours although no analysis of ER was attempted in this study (Zapata et al, 1998). The highly variable expression of BAG-1 in breast cancers may be a useful prognostic/predictive marker. For example, not all ER-positive tumours respond to hormone therapy and it is possible that analysis of BAG-1 expression, in addition to other markers of ER function, may be of benefit in predicting response of individual tumours to hormone therapy. BAG-1 may also be important for growth of tumours via direct effect of ER or BCL-2-dependent suppression of apoptosis and BAG-1 may be an attractive target for therapeutic intervention in tumours. Although further studies are required to address these issues, breast cancer cells provide a biologically relevant system to dissect the functional roles of different BAG-1 isoforms.

### ACKNOWLEDGEMENTS

We are very grateful to Sue Davies for technical support and thank Drs Dutrillaux and Hurst for providing cell lines. This work was supported by the Ludwig Institute for Cancer Research.

### REFERENCES

Adachi M, Masuo S, Torigoe T, Takayama S, Reed JC, Miyazaki T, Minami Y, Taniguchi T and Imai K (1996) Interleukin-2 (IL-2) upregulates Bag-1 gene expression through serine-rich region within IL-2 receptor βc chain. *Blood* **88**: 4118–4123

Bardelli A, Longati P, Albero D, Goruppi S, Schneider A, Ponzetta C and Comoglio PM (1996) HGF receptor associates with the anti-apoptotic protein Bag-1 and prevents cell death. *EMBO J* 15: 6205–6212

Bardon S, Vignon F, Montcourrier P and Rochefort H (1987) Steroid receptor-mediated cytotoxicity of an antiestrogen and an antiprogestin in breast cancer cells. *Cancer Res* **47**: 1441–1448

Beveglia L, Matsumoto K, Lin CS, Ziober BL and Kramer RH (1997) Expression of the c-Met/HGF receptor in human breast carcinoma; correlation with tumor progression. *Int J Cancer* **74**: 301–309

Bhargava V, Kell DL, van de Rijn M and Warnke RA (1994) Bcl-2 immunoreactivity in breast carcinoma correlates with hormone receptor positivity. *Am J Pathol* **145**: 535–540

Chalbos D, Vignon F, Keydar I and Rochefort H (1982) Estrogens stimulate cell proliferation and induce secretory proteins in a human breast cancer cell line (T47D). *J Clin Endocrinol Metab* **55**: 276–283

Clarke C, Titley J, Davies S and O'Hare MJ (1994) An immunomagnetic separation method using supermagnetic (MACS) beads for large-scale purificiation of human mammary luminal and myoepithelial cells. *Epithelial Cell Biol* **3**: 38–46

Clevenger CV, Thickman K, Ngo W, Chang W-P, Takayama S and Reed JC (1997) Role of Bag-1 in the survival and proliferation of the cytokine-dependent lymphocyte lines, Ba/F3 and Nb2. *Mol Endocrinol* **11**: 608–618

Coradini D, Cappelletti V, Granta G and di Fonzo G (1991) Activity of tamoxifen and its metabolites on endocrine-dependent and endocrine-independent breast cancer cells. *Tumour Biol* **12**: 149–158

Engel LW, Young NA, Tralka TS, Lippman ME, O'Brien SJ and Joyce MJ (1978) Establishment and characterisation of three new continuous cell lines derived from human breast carcinomas. *Cancer Res* **38**: 3352–3364

Fredersdorf S, Milne AW, Hall PA and Lu X (1996) Characterisation of a panel of novel anti-p21waf1/cip1 monoclonal antibodies and immunochemical analysis of p21waf1/cip1 expression in normal human tissues *Am J Pathol* **148**: 825–835

Froesch BA, Takayama S and Reed JC (1998) Bag-1L protein enhances androgen receptor function. *J Biol Chem* **273**: 11660–11666

Gioanni J, Le FD, Zanghellini E, Mazeau C, Ettore F, Lambert JC, Schneider M and Dutrillaux B (1990) Establishment and characterisation of a new tumorigenic cell line with a normal karyotype derived from a human breast adenocarcinoma *Br J Cancer* **62**: 8–13

Hohfeld J and Jentsch S (1997) GrpE-like regulation of the hsc70 chaperone by the anti-apoptotic protein Bag-1 *EMBO J* **16**: 6209–6216

Huang Y, Ray S, Reed JC, Ibrado AM, Tang C, Nawabi A and Bhalla K (1997) Estrogen increases intracellular p26Bcl-2 to p21 Bax ratios and inhibits taxol-induced apoptosis of human breast cancer MCF-7 cells. *Breast Cancer Res Treat* **42**: 73–81

Hurst J, Maniar N, Tombarkiewicz J, Lucas F, Roberson C, Steplewski Z, James W and Perras J (1993) A novel model of a metastatic human breast tumour xenograft line. *Br J Cancer* **6**: 274–276

Kullmann M, Schneikert J, Moll J, Heck S, Zeiner M, Gehring U and Cato ACB (1998) Rap46 is a negative regulator of glucocorticoid receptor action and hormone-induced apoptosis. *J Biol Chem* **273**: 14620–14625

Kyprianou N, English HF, Davidson NE and Isaacs JT (1991) Programmed cell death during regression of the MCF-7 human breast cancer following estrogen ablation. *Cancer Res* **51**: 162–166

Landel CC, Kushner PJ and Greene GL (1994) The interaction of human estrogen receptor with DNA is modulated by receptor associated proteins. *Mol Endocrinol* **8**: 1407–1419

Leek RD, Kaklamanis L, Pezzella F, Gatter KC and Harris AL (1994) Bcl-2 in normal human breast and carcinoma, association with oestrogen receptor-positive, epidermal growth factor receptor-negative tumours and in situ cancer. *Br J Cancer* **69**: 135–139

Lippman ME and Dickson RB (1989) Mechanisms of growth control in normal and malignant breast epithelium. *Recent Prog* **45**: 383–440

Lippman ME, Bolan G and Huff K (1976) The effects of estrogens and anti-estrogens on hormone-responsive human breast cancer in long-term tissue culture. *Cancer Res* **36**: 4595–4601

Liu R, Takayama S, Zheng Y, Froesch B, Chen G-Q, Zhang X, Reed JC and Zhang X-K (1998) Interaction of BAG-1 with retinoic acid receptor and its inhibition of retinoic acid-induced apoptosis in cancer cells. *J Biol Chem* **273**: 16985–16992

Matsuzawa S-I, Takayama S, Froesch BA, Zapata JM and Reed JC (1998) p53-inducible human homologue of drosophila seven in absentia (siah) inhibits cell growth: suppression by Bag-1. *EMBO J* **17**: 2736–2747

Packham G, Brimmell M and Cleveland JL (1997) Mammalian cells express two differently localized BAG-1 isoforms generated by alternate translation. *Biochem J* **328**: 807–813

Reed JC (1997) Double identity for proteins of the Bcl-2 family. *Nature* **387**: 773–776

Schulz JB, Bremen D, Reed JC, Lommatzsch J, Takayama S, Wullner U, Loschmann PA, Klockgether T and Weller M (1997) Cooperative interception of neuronal apoptosis by bcl-2 and bag-1 expression; prevention of caspase activation and reduced production of reactive oxygen species. *J Neurochem* **69**: 2075–2086

Silvestrini R, Veneroni S, Daidone MG, Benini E, Boracchi P, Mezzetti M, di Fronzo G, Rilke F and Veronesi U (1994) The Bcl-2 protein: a prognostic indicator strongly related to p53 protein in lymph node-negative breast cancer patients. *J Natl Cancer Inst* **86**: 499–504

Takayama S, Sato T, Krajewski S, Kochel K, Irie S, Millan JA and Reed JC (1995) Cloning and functional analysis of Bag-1: a novel Bcl-2-binding protein with anti-cell death activity. *Cell* **80**: 279–284

Takayama S, Kochel K, Irie S, Inazawa J, Abe T, Sato T, Druck T, Huebner K and Reed JC (1996) Cloning of cDNAs encoding the human BAG1 protein and

 © 1999 Cancer Research Campaign localisation of the human BAG1 gene to chromosome 9p12. *Genomics* **35**: 494–498

Takayama S, Bimston DN, Matsuzawa S, Freeman BC, Aime-Sempe C, Xie Z, Morimoto RJ and Reed JC (1997) Bag-1 modulates the chaperone activity of hsp70/hsc70. *EMBO J* **16**: 4887–4896

Takayama S, Krajewski S, Krajewski M, Kitada S, Zapata JM, Kochel K, Knee D, Scudiero D, Tudor G, Miller GJ, Miyashita T, Yamada M and Reed JC (1998) Expression and location of HSP70/HSC-binding anti-apoptotic protein BAG-1 and its variants in normal tissues and tumor cell lines. *Cancer Res* **58**: 3116–3131

Teixeira C, Reed JC and Pratt MAC (1995) Estrogen promotes chemotherapeutic drug resistance by a mechanism involving bcl-2 proto-oncogene expression in human breast cancer cells. *Cancer Res* **55**: 3902–3907

Tuck AB, Park M, Sterns EE, Boag A and Elliott BE (1996) Coexpression of hepatocyte growth factor and receptor (met) in human breast carcinoma. *Am J Pathol* **148**: 225–232

Wakeling AE, Dukes M and Bowler J (1991) A potent specific pure antiestrogen with clinical potential. *Cancer Res* **51**: 3867–3873

Wang HG, Takayama S, Rapp UR and Reed JC (1996) Bcl-2 interacting protein, Bag-1, binds to and activates the kinase Raf-1. *Proc Natl Acad Sci USA* **93**: 7063–7068

Wilson JW, Wakeling AE, Morris ID, Hickman JA and Dive C (1995) MCF-7 human mammary adenocarcinoma cell death in vitro in response to hormone-withdrawal and DNA damage. *Int J Cancer* **61**: 502–508

Zapata JM, Krajewska M, Krajewska S, Huang R-P, Takayama S, Wang H-G, Adamson E and Reed JC (1998) Expression of multiple apoptosis-regulatory genes in human breast cancer cell lines and primary tumours. *Breast Cancer Res Treat* **47**: 129–140

Ziener M and Gehring U (1995) A protein that interacts with members of the nuclear hormone receptor family: identification and cDNA cloning. *Proc Natl Acad Sci USA* **92**: 11465–11469

Zeiner M, Gebauer M and Gehring U (1997) Mammalian protein RAP46: an interaction partner and modulator of 70 kDa heat shock proteins. *EMBO J* **167**: 5483–5490

*© 1999 Cancer Research Campaign*
